# Feasibility, Acceptability, and Preliminary Impact of an Online Latina Mother–Daughter Physical Activity Intervention

**DOI:** 10.1123/jpah.2025-0646

**Published:** 2026-04-28

**Authors:** Elva M. Arredondo, Jennifer L. Schneider, Marisa S. Torres-Ruiz, Victoria M. Telles, Katie Butte, Oliva Lafuente, Taynara Formagini, Athena Cisneroz, Scott C. Roesch, Tom Baranowski, Guadalupe X. Ayala

**Affiliations:** 1 Department of Psychology, San Diego State University, San Diego, CA, USA; 2 Institute for Behavioral and Community Health (IBACH), San Diego State University Foundation, San Diego, CA, USA; 3 Joint Doctoral Program in Public Health at San Diego State University-University of California, San Diego, CA, USA; 4 Department of Health and Human Performance, Seattle Pacific University, Seattle, WA, USA; 5 Department of Family Medicine, University of California San Diego, San Diego, CA, USA; 6 Pediatrics-Nutrition, USDA Funded Children’s Nutrition Research Center, Baylor College of Medicine, Houston, TX, USA; 7 School of Public Health, San Diego State University, San Diego, CA, USA

**Keywords:** cultural adaptation, family-based health promotion, dyadic analysis, parenting practices, health equity

## Abstract

**Background:**

Latina girls have low rates of moderate to vigorous physical activity (PA) compared with their male counterparts and non-Hispanic White girls. The current study examines the feasibility, acceptability, and preliminary impact of *Conmigo*, a mother–daughter PA intervention for preadolescent Latinas (ages 8–11) delivered online.

**Methods:**

Seventy-nine mother–daughter dyads were recruited. Dyads were randomized to a PA intervention or a delayed, abbreviated control condition. The PA intervention consisted of 12 weekly 1.5-hour virtual PA sessions. Feasibility was measured by the delivery, receipt, and enactment of the intervention; acceptability was measured by satisfaction in surveys and interviews in the PA intervention condition. PA and associated secondary outcomes were measured at baseline and follow-up (12 wk) using accelerometry and self-report.

**Results:**

Most mothers had some college education (63.3%), and 44.3% worked full time; most daughters were US-born (91.1%) compared with mothers (38.0%). Program fidelity was high (81%–95%) with a mean attendance of 6.2 (SD = 4.6) across 12 sessions. Satisfaction was high (mean = 4.6, SD = 0.8). Qualitative findings indicated that the curriculum met families’ needs. No significant changes were observed in daughters’ accelerometer-assessed or self-reported PA. A marginal improvement (*B* = 0.37, *P* < .05) was found in limit-setting behavior; other parenting outcomes and dyadic effects were nonsignificant.

**Conclusions:**

Qualitative and quantitative findings were not fully aligned regarding feasibility, acceptability, and preliminary impact of the online *Conmigo* program. Nonetheless, online mother–daughter PA interventions may represent a scalable strategy to have high reach among Latino families.

Only 26.1% of US adolescents meet current physical activity (PA) guidelines.^1^ Fewer girls (17.5%) meet the guidelines than boys (35.3%).^2^ These trends continue into adulthood with a pronounced decline in girls’ PA during adolescence,^3^ due to the biological, mental, and social changes during puberty.^4-6^ Intervening during the prepubertal phase (typically between ages 8 and 11 y old) and targeting factors that influence PA in girls can help curb the steep drop-off in PA during adolescence and shape girls’ PA behaviors for adulthood.^7,8^ Sociocultural factors including immigrant experiences, family expectations, acculturation, dietary practices, and other environmental factors explain, in part, disparities in PA.^9-12^

Guided by Social Cognitive Theory^13^ and Family Systems Theory,^14^ PA is conceptualized as a behavior shaped by reciprocal interactions among individual, interpersonal, and contextual factors. These frameworks emphasize modeling, social support, self-regulation, and bidirectional influences within families, providing a strong theoretical rationale for dyadic interactions and parenting processes as mechanisms of behavior change. Empirical evidence supports this perspective, showing that mothers’ PA and PA-related parenting practices are associated with their daughters’ PA.^15-17^ Although family influences are often hierarchical—frequently operating from mothers to daughters—parenting is also responsive to children’s traits, behaviors, and broader social contexts.^18,19^ As a result, children can influence their parents’ PA both directly and indirectly through encouragement, support, and shared activity.^20,21^ In addition, family-level processes such as parent–child communication have been shown to promote PA and other health behaviors among girls.^22^ Taken together, these findings suggest that interventions that intentionally leverage family dynamics and bidirectional processes may be particularly effective in promoting PA among children.

More recently, virtually delivered, parent-focused intervention modalities have emerged as a compelling approach to promoting healthy lifestyle behaviors^23^ and may be particularly advantageous for diverse and underserved families who face structural and logistical barriers to participation in in-person programs, including time constraints, transportation limitations, and competing family and caregiving responsibilities.^24^ Virtually delivered, group-based interventions also offer opportunities to enhance reach, scalability, and cost-effectiveness.^25^ Despite these potential advantages, relatively few studies have systematically evaluated the feasibility and acceptability of virtually delivered, group-based PA interventions, and, to our knowledge, none have examined the feasibility and acceptability of mother–daughter interventions delivered virtually among Latino communities. As a result, critical gaps remain in understanding whether virtually delivered, family-based interventions can be successfully implemented and sustained, particularly among populations experiencing health disparities.

Systematic reviews suggest that mother–daughter interventions are a promising strategy for increasing daughters’ PA by leveraging familial relationships to support behavior change.^26,27^ However, prior research has highlighted the need for culturally tailored, theory-based mother–daughter interventions delivered remotely or in the family home, beyond single-arm designs and studies relying solely on device-based PA measures. This pilot study addressed these gaps by evaluating the feasibility, acceptability, and preliminary impact of a mother–daughter PA intervention (Conmigo [With Me]) on daughters’ moderate to vigorous physical activity (MVPA), assessed using both self-report and accelerometer methods. The primary aims were to assess feasibility and acceptability, with secondary aims examining preliminary impact on daughters’ MVPA and hypothesized mechanisms of change, including mothers’ MVPA, PA-related parenting practices, mother–daughter communication, and bidirectional PA influences. We hypothesized that Conmigo would be feasible and acceptable, increase daughters’ MVPA, and improve mothers’ PA and PA-related parenting practices, and that improvements in mother–daughter communication and co-participation in PA would be associated with increased daughters’ MVPA. This pilot was designed to inform the design and powering of a future randomized controlled trial.

## Methods

### Study Design

The current study was designed as a 2-arm randomized controlled trial assessing the acceptability, feasibility, and preliminary impact of a culturally tailored mother–daughter intervention to increase MVPA among dyads living in San Diego County. Latina preadolescents and their mothers were randomized using a simple randomization approach with a 1:1 allocation ratio. Specifically, each dyad was independently assigned to either the intervention or delayed control condition using a computer-generated random assignment equivalent to a coin flip, with no blocking or stratification. *Conmigo* was originally proposed as an in-person intervention, but was developed for online delivery during the initial phase due to the COVID-19 pandemic. The study was approved by the Institutional Review Board of San Diego State University Human Research Protection Program and funded through the National Institute of Child Health and Human Development (ClinicalTrials.gov Identifier: NCT04736030).

### Participants—Recruitment and Eligibility Criteria

Latina mother–daughter dyads were recruited from community organizations that serve Latinos, including libraries, churches, elementary schools, and advocacy groups. Due to pandemic restrictions, recruitment and screening were conducted online (eg, Zoom presentations in virtual classrooms) and by mail (eg, flyers distributed by community partners). Interested mothers completed an initial eligibility screening through Qualtrics XM,^28^ which featured a study overview video describing the intervention, research design, and participant commitments. After viewing the video, mothers completed an eligibility questionnaire. A staff member contacted eligible dyads to complete a second screening via phone, which consisted of the Physical Activity Readiness Questionnaire^29^ to confirm their ability to safely engage in PA.

Participant eligibility required: (1) daughters aged 8–11 years not meeting the Centers for Disease Control’s 2018 PA guidelines (ie, <60 min MVPA/d), (2) mothers as primary caregivers (≥4 d/wk), (3) both mother and daughter self-identified as Latina, (4) ability to attend weekly 90-minute sessions for 12 weeks, (5) intention to remain in the area during the study, and (6) access to internet-connected devices. All eligibility criteria were self-reported by mothers in a screening questionnaire. Dyads were excluded if either had a health condition limiting PA or could not complete consent in English or Spanish; mothers or daughters with health risk factors per the Physical Activity Readiness Questionnaire were required to provide medical clearance before enrolling. For families with multiple eligible daughters, one was randomly selected.

**Figure 1 —:**
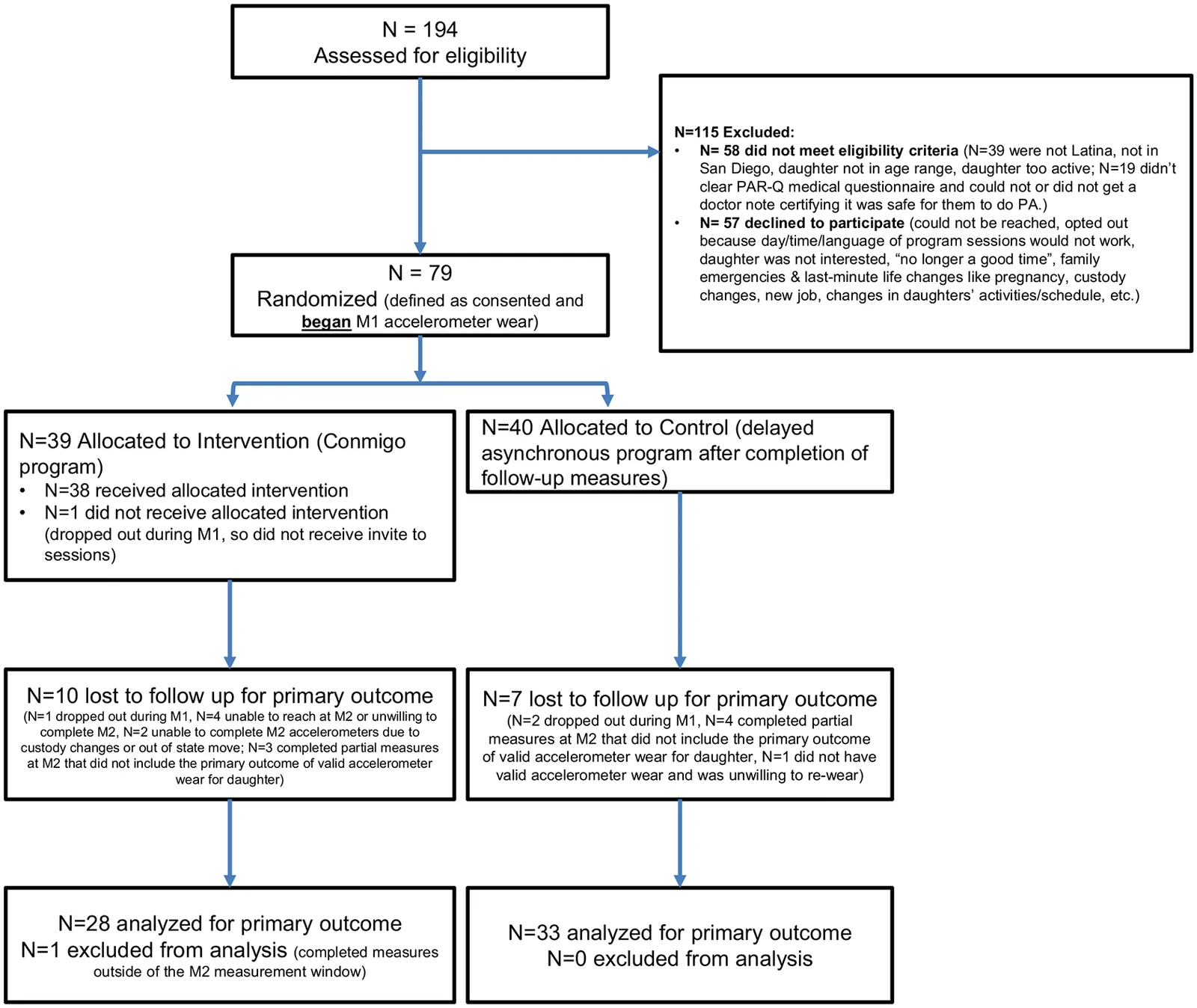
CONSORT diagram of recruitment and dyad allocation. Questionnaire. PA indicates physical activity; PAR-Q, Physical Activity Readiness Questionnaire.

Groups of up to 20 eligible dyads attended a Zoom orientation describing study commitments and logistics (eg, randomization). Following orientation, mothers provided consent via phone, and daughters provided assent. Following consent and confirmation of accelerometer wear, dyads were randomized to intervention or control (up to 10 in each group). We capped each intervention group at 10 dyads to balance logistical feasibility for Zoom-based delivery with group dynamics that support meaningful interaction and effective facilitation, consistent with prior group-based intervention literature.^30^ Of 194 dyads screened, 79 (39 intervention, 40 control) were enrolled. Details of the power calculations are provided in the protocol paper.^31^ Figure 1 outlines recruitment and allocation.

#### PA Intervention

*Conmigo* is a culturally tailored intervention to improve the mother–daughter relationship and their health behaviors. It uses a holistic approach and unique strategies to target key parent and family-level factors relevant to Latina mothers and daughters to motivate and sustain PA. The intervention is informed by Social Cognitive^13,32,33^and Family Systems Theories,^14^ and culturally adapted for Latino families. Extensive details of how the theory aligns with the intervention components can be found in the protocol paper.^31^ The intervention aims to empower Latina mothers by providing them with information and practical skills related to child development, the challenges of adolescence, and the unique hurdles many immigrant families face when navigating different cultural contexts. Specifically, *Conmigo* seeks to promote co-participation in PA among mothers and daughters, improve mothers’ ability to connect with their children, facilitate better family communication, refine parenting practices related to PA and nutrition (eg, monitoring, limit setting), and equip preadolescents with the skills needed to face adverse negative influences during this life stage, including those experienced by exposure to social media. Details about the curriculum can be found in the protocol paper.^31^

In response to the COVID-19 pandemic, the intervention was developed for delivery via Zoom. Activities were developed for online engagement, with input from an elementary education specialist and a Community Advisory Board of Latino parents and educators to ensure cultural and developmental relevance. Sessions were co-led by a certified PA instructor and a research team member, with a research assistant collecting process data.

The 30-minute group exercise sessions (eg, cardio kickboxing, dance, bodyweight training using household items) were combined with 60-minute didactic components, including brief check-ins, culturally tailored lessons (eg, communication, parenting, screen time), participatory activities, and goal setting. Sessions included joint mother–daughter and separate formats. Physical activities were designed to enhance self-efficacy and were adapted for all fitness levels. Cultural relevance was emphasized through music, visuals, bilingual delivery, and integration of Latino family and dietary norms. Weekly PA challenges reinforced Social Cognitive Theory constructs (eg, skill-building, social support), with dyads earning points for participation and sharing reflections via a closed WhatsApp group.

### Evaluation

#### Aim 1: Feasibility and Acceptability

##### Feasibility.

Feasibility was assessed using the National Institute of Health Behavioral Change Consortium Treatment Fidelity Framework, focusing on intervention delivery, receipt, and enactment.^34^ Data collection involved self-reported and observational methods.

##### Intervention Delivery.

Delivery was assessed through *dose delivered* and *fidelity*. The dose delivered included the number of scheduled and completed sessions (out of 12 possible). Fidelity was evaluated using an adapted version of Breitenstein’s Fidelity Checklist, with didactic and PA facilitators completing a 10- to 16-item self-assessment for each session.^35^ Didactic facilitators documented the completion of each subsection of the curriculum, including welcoming participants, leading icebreakers, addressing barriers to PA, presenting topics, and guiding group activities. PA instructors assessed the degree to which they completed actions such as demonstrating exercise modifications and providing feedback on participants’ form. Facilitators rated adherence to session protocols on a scale of “yes,” “somewhat,” or “no” indicating full, partial, or no coverage of expected activities and behaviors.

##### Intervention Receipt.

Receipt was measured through *dose received*, which was calculated as participant attendance across intervention waves relative to dose delivered.

##### Enactment of Intervention Skills.

Enactment was assessed through self-reported performance of intervention skills. After each session, participants reported the average number of days per week they engaged in PA outside of sessions and the frequency with which they practiced behavioral recommendations, such as communication skills, parenting strategies, and dietary changes.

#### Acceptability

Program acceptability was assessed using postintervention surveys and semistructured interviews. Surveys captured overall program satisfaction as reported by the mothers, while interviews explored mothers’ and daughters’ experiences, focusing on acceptability of content and delivery, and barriers and facilitators to participation. At the end of the 12-week intervention, all dyads randomized to the intervention condition were invited to participate in separate interviews in their preferred language (Spanish/English). Interviews lasted 30 to 60 minutes for mothers and 20 to 30 minutes for daughters. The interview guides included probes to assess satisfaction with program components (eg, goal setting, weekly check-ins, group PA) and topics (eg, communication, parenting, puberty), feedback on the program length and the online format, and the likelihood that they would recommend the program to others.

#### Aim 2: Preliminary Impact on Primary and Secondary Outcomes

##### Quantitative Data Collection.

Participants completed objective and self-report measures of PA (primary outcome) and self-report questionnaires for secondary outcomes. Surveys were available in English and Spanish, using validated translations or those reviewed by bilingual staff. Questionnaires were administered via Qualtrics^28^; mothers completed them online or by phone, while daughters completed them by phone with staff support. Mother surveys took 30 to 60 minutes to complete, and daughter surveys took 30 to 40 minutes. Follow-up interviews also explored the perceived impact of the program on primary and secondary outcomes.

##### Physical Activity (Primary Outcome).

PA levels for mothers and daughters were assessed at baseline and follow-up using ActiGraph GT3X+ accelerometers. Devices were worn on the right hip during waking hours for 7 consecutive days, with wear time validated using the Choi algorithm (≥3 d, ≥10 h/d). MVPA was calculated using standard cut points (≥1952 counts/min for mothers; ≥2296 for daughters).^36,37^ Daughters’ self-reported PA was assessed using the PA domain of the Family Life, Activity, Sun, Health, and Eating (FLASHE) questionnaire.^38^ We included items on weekday and weekend PA levels (eg, “How much PA did you do last Saturday?”), with response options on a 5-point Likert scale from 0 (never) to 4 (4–5 d/wk or mostly every day). Depending on school attendance (in-person or online), daughters completed 8 or 11 questions.

#### Secondary Outcomes

##### Mother–Daughter Communication.

Mothers completed the Parent-Adolescent Communication Scale to assess mother–daughter communication.^39^ The Parent-Adolescent Communication Scale Spanish version was originally available through the Pan American Health Organization. The measure was reviewed by bilingual and bicultural members of our research team to ensure cultural and linguistic appropriateness and contains 20 items using a 5-point Likert scale (*α* = .80 for openness and *α* = .67 for problems). The original measure was revised to use “daughter” as the referent (eg, My daughter is always a good listener).

##### Parenting Strategies for Healthy Eating and PA.

Mothers completed the Parenting for Eating and Activity Scale, which was developed and validated in a Spanish-speaking sample.^40^ Mothers completed 3 of the 5 subscales with items related to both PA and nutrition: limit setting (6 items; *α* = .86), monitoring (7 items; *α* = .85), and reinforcement (2 items; *r* = .61). Responses were provided on a 5-point Likert scale from 1 = disagree to 5 = agree. The measure was revised to use “daughter” as the referent.

Mothers’ self-reported PA was measured using the Global Physical Activity Questionnaire (GPAQ) developed by the World Health Organization.^41^ The GPAQ assessed PA across 3 domains (occupation, transportation, and leisure-time). Across domains, the GPAQ captured PA frequency (days a week), duration (hours or minutes), and intensity (light, moderate, or vigorous) through 16 items. Four additional questions were administered to capture MVPA and strength training. The measure was validated with Spanish-speaking Latinas by our research team.^42^

##### Sociodemographic Characteristics.

Mothers reported their sociodemographic characteristics, including age, country of birth, marital status, education level, employment status, family monthly income, and the age and country of birth of their daughters. Preferred survey language for both mothers and daughters was also recorded.

##### Qualitative Data Collection.

Qualitative data were collected through postintervention interviews in which staff asked mothers to reflect on the impact of the program on their PA behaviors, dietary habits, and the quality of their relationships with their daughters. These interviews aimed to capture the mothers’ perspectives on how the program influenced their health behaviors and strengthened family dynamics, as well as program feasibility and acceptability.

#### Data Analyses

##### Aims 1 and 2: Qualitative Analysis.

We utilized a rapid qualitative approach to analyze the postintervention interviews.^43,44^ Each interview was transcribed verbatim in English or Spanish, translated to English (if applicable), and then summarized using a 2-page template of all question constructs. Summaries were entered into a matrix by construct, and research staff conducted topic monitoring to determine salient themes and codes across all interviews. Mother–daughter interviews were analyzed separately using distinct summary templates and matrices.

##### Aims 1 and 2: Quantitative Analyses.

All data were reviewed for completeness and accuracy before analysis. Descriptive data (eg, means and frequencies) were used to assess the feasibility and acceptability of the study following its completion. During the data screening phase, we examined data distributions for normality and used the full-information maximum likelihood approach for missing data, as implemented in MPlus. Assessment of outcomes followed the intention-to-treat principle. Mothers’ and daughters’ ages were evaluated as potential covariates in these models. Multilevel (mixed-effects) modeling was used as the primary statistical model due to the nested structure of the data: (1) repeated measures (time) [level 1] nested within dyads [level 2]. Of primary interest in these analyses is the Time X Treatment Group cross-level interaction effect. Statistically significant interactions were followed up with simple slope analyses as outlined by Preacher et al.^45^

Dyadic analyses were used to account for the interdependence of mothers’ and daughters’ outcomes, consistent with the intervention’s design and theoretical emphasis on co-participation and mutual influence. In the context of multilevel models, actor and partner effects (as separate predictor variables) were modeled simultaneously so that the bidirectional influence of the relationships of interest could be accounted for. Using this standard design over time, the dyadic data analytic approach followed the recommendations of Brown et al^46^ and Kenny and Kashy,^47^ and the Prevention Science and Methodology Group for randomized controlled trials.

## Results

Figure 1 depicts the CONSORT diagram.

### Sociodemographic Characteristics

Table 1 depicts participants’ sociodemographic characteristics. The sample consisted of mothers with a mean age of 38.7 years (SD = 6.3) and daughters with a mean age of 9.8 years (SD = 1.2). Most mothers (74.7%) were married or living as married. Most daughters (91.1%) were born in the United States, while 38.0% of mothers were US-born. Most mothers (44.3%) were employed full time; the family’s monthly income was distributed across 3 categories, and 63% of the mothers had at least some college education.

### Aim 1: Feasibility and Acceptability

Table 2 presents descriptive data assessing the feasibility and acceptability of the intervention across multiple domains. The program was delivered with good fidelity (81%–95%) but had moderate dose delivered (6.2 attendance out of 12). There was high satisfaction. To complement these quantitative findings, we asked questions during the postintervention interviews with mothers and daughters to gather qualitative insights into the program’s acceptability. Of the 39 dyads invited, 16 mothers and 16 daughters (n = 32) agreed to participate in the postintervention interviews. Findings from the interviews revealed that the program was well received by mothers and daughters, with participants expressing satisfaction with the curriculum and its alignment with their family values (see Table 3). The online delivery format helped reduce logistical barriers like childcare and transportation; however, many participants preferred in-person sessions for greater engagement and social interaction. Some participants also reported technical difficulties, such as internet disruptions. Overall, mothers appreciated the program’s positive impact on their relationships and health behaviors and expressed willingness to recommend it to others.

### Aim 2: Preliminary Impact on Primary and Secondary Outcomes

#### Multilevel Modeling Analyses of Primary and Secondary Outcomes; Dyadic Analyses.

##### Primary Outcome of PA.

Although there was a trend toward increased MVPA over time among daughters in the PA intervention condition, this did not reach statistical significance. Similarly, the condition × time interaction for self-reported PA (FLASH Z score) was not significant, although a moderate effect size was observed (Cohen *d* = 0.48). For mothers, the condition × time interaction for daily minutes of MVPA was not statistically significant for both accelerometer and self-reported PA (GPAQ).

##### Secondary Outcomes of Parenting and Communication.

There was a marginally significant condition × time interaction for limit setting (*B* = 0.37, *P* < .05), with a medium effect size (Cohen *d* = 0.52), suggesting a potential improvement in limit setting of unhealthy behaviors among mothers in the PA intervention condition. The interaction for communication was not significant, although it had a moderate effect size (Cohen *d* = −0.39). No significant condition × time interactions were found for monitoring or reinforcement, with very small effect sizes.

##### Dyadic Analyses.

There were no significant dyadic associations observed between mothers and daughters on any of the primary or secondary outcome measures.

Qualitative findings showed the program impacted participants’ PA and dietary behaviors. Mothers reported that the program impacted their parenting practices. Mothers and daughters reported engaging in more PA together and incorporating small but dietary changes. Participants highlighted improved mother–daughter communication and stronger relationships as key facilitators of behavior change, as well as support from weekly challenges that fostered accountability and motivation to stay active (Table 4).

## Discussion

This study evaluated the feasibility, acceptability, and preliminary impact of an online, culturally tailored mother–daughter PA intervention for Latina preadolescents and their mothers. Feasibility-related findings suggest that delivering a mother–daughter PA intervention is a promising approach to engaging Latino families in behavior change together. Facilitators demonstrated strong fidelity to PA and content delivery, and participants reported practicing the topics outside of class. Each session was delivered as planned; however, on average, mothers and daughters attended only about half of the sessions. These attendance patterns highlight important considerations for the design of future large-scale trials, including the need to optimize intervention dose, reduce participant burden, and incorporate strategies to improve retention and sustained engagement.

Acceptability was generally high, as mothers and daughters reported enjoying the program activities, noting positive changes in their mother–daughter relationships, and expressing their willingness to recommend the program to others. However, challenges in delivering the intervention virtually include technological issues (eg, poor internet connection) and difficulty keeping young girls engaged online (eg, due to Zoom fatigue). Research shows that technological issues and the use of technologies not integrated into daily life (eg, desktop computers) inhibit engagement in digital health interventions among children and young people.^48^ For future virtual interventions, it will be essential to address these challenges by developing innovative strategies that better engage participants and address technological barriers. Nonetheless, many mothers would have preferred in-person programming to facilitate connections with other families and better engage their daughters.

Quantitative data show no significant changes in daughters’ accelerometer-assessed or self-reported PA postintervention; however, there were trends in the expected direction and a moderate effect size for self-reported PA. Qualitative findings highlight positive changes in health behaviors among mothers and daughters, including increased exercise, healthier dietary habits, and enhanced mother–daughter interactions around PA. Consistent with our study findings, evidence for the effectiveness of mother–daughter interventions to improve PA is still emerging, with studies showing mixed effects. Previous pilot studies have shown mixed results. One nonrandomized trial with 46 Latina mother–daughter dyads found improved aerobic capacity in daughters but no change in accelerometer-assessed MVPA or mothers’ PA.^49^ Similarly, an after-school intervention with 76 African American dyads reported increased vigorous PA but no change in daughters’ total daily PA.^50^

Moreover, we found no significant effects on hypothesized mechanisms of change. Although the program aimed to strengthen mother–daughter communication and parenting practices, these changes were not reflected in the quantitative measures, and no bidirectional effects were observed between mothers’ and daughters’ PA. In contrast, qualitative findings indicated improved communication and strengthened relational bonds. Several factors may explain the lack of quantitative change. Parenting behaviors are often habitual and may require more intensive or prolonged intervention to shift. Additionally, the communication scale and some parenting measures—such as those focused on sticker charts and behavior tracking—may not have aligned well with the intervention targets and Latino cultural practices, potentially limiting their relevance and sensitivity to change.^51^ We also found that the children (particularly those who were 8–9 y old) had more difficulty responding to several of the parenting and communication measures. These findings suggest that future culturally tailored family-based interventions may benefit from greater use of culturally responsive and developmentally appropriate measures of parenting and family dynamics.

This study had several limitations, including a small sample size that limited statistical power to detect significant associations and bidirectional effects, and differential attrition between the intervention and control groups, which may introduce attrition bias and limit interpretation of quantitative trends. Although attrition was not associated with measured sociodemographic characteristics, post hoc analyses suggest that participants with poor intervention attendance were less likely to complete follow-up assessments compared with those attended regularly. Despite these limitations, this pilot study provides valuable evidence on the feasibility, acceptability, and potential mechanisms of a culturally responsive, remotely delivered mother–daughter PA intervention, informing the design of a fully powered randomized controlled trial. The current study also had several notable strengths. First, participants’ PA was measured objectively using accelerometry. Second, the intervention was culturally tailored to Latina mothers and daughters, considering cultural preferences and activities, and delivered by bilingual/bicultural staff, which may have enhanced the program’s relevance for this population and its ability to reach dyads of various acculturation levels. Finally, the collection of both qualitative and quantitative data for feasibility, acceptability, and program impact allowed for a more comprehensive analysis of study outcomes.

Online programs may enhance accessibility, particularly for individuals in rural areas or those with transportation and childcare challenges, by offering flexible engagement. However, qualitative and quantitative findings suggest that in-person formats may have greater reach and impact among marginalized communities. The digital divide^52^—including limited internet access, low technology literacy, and lack of privacy—continues to hinder online participation. Despite these limitations, online formats remain promising due to their scalability and lower resource demands.

Findings from this pilot study inform the design of future large-scale trials by demonstrating the feasibility of mother–daughter co-participation and highlighting the need for strategies to improve attendance and retention, including hybrid delivery models, reduced session burden, and enhanced technological support. Observed trends in PA and discrepancies between quantitative and qualitative outcomes also provide guidance for outcome selection, measurement alignment, and power calculations. Beyond methodological implications, this study underscores the importance of culturally tailored, family-based interventions that foster joint participation among Latina mothers and daughters to strengthen relationships and promote healthy behaviors.^53^ The culturally responsive design of Conmigo—including bilingual/bicultural facilitation, relationship-centered content, and attention to differences in mother and daughter acculturation levels—aligns with recent evidence supporting the acceptability of virtual parent–child PA interventions for diverse communities.^54^ From a public health perspective, these findings suggest that family-based, Latino tailored PA interventions are promising approaches to addressing PA disparities in Latino communities, while also emphasizing key implementation considerations such as the digital divide and the potential benefits of in-person or hybrid delivery models. Collectively, these results position Conmigo as an adaptable model to inform future research, practice, and policy efforts.

## Figures and Tables

**Table 1:** Sociodemographic Characteristics

	Mean (SD) or n (%)
Age, mean (SD)	
Mothers	38.7 (6.3)
Daughters	9.9 (1.2)
English language preference for survey, n (%)	
Mothers	54 (68.4)
Daughters	60 (74.1)
US country of birth, n (%)	
Mothers	30 (38.0)
Daughters	72 (91.1)
Mothers’ marital status, n (%)	
Married or living as married	59 (74.7)
Not married	19 (24.1)
Mothers’ education, n (%)	
High school or less	28 (35.5)
Some college (1–4 y)	26 (32.9)
College graduate/postgraduate	24 (30.4)
Mothers’ employment status, n (%)	
Full time	35 (44.3)
Part time	10 (12.7)
Homemaker/unemployed	33 (41.7)
Family monthly income, n (%)	
≤$1500	10 (12.7)
$1501–3000	27 (34.1)
≥$3001	27 (34.2)

Note: There were no sociodemographic differences between mothers and daughters in the intervention versus control conditions.

**Table 2:** Feasibility and Acceptability of Intervention (n = 32)

Feasibility		
Delivery	Dose delivered (of 12 planned sessions)	12
	Instructor fidelity for didactic content delivery	95.0%
	Instructor fidelity for PA	81.0%
Receipt	Dose received—average participant attendance of 12 sessions	6.2 (4.6)
Enactment^a^	Performance of PA skills outside of sessions each week	2.5 (1.7)
	Practice recommendations outside of sessions (parenting, nutrition, and communication)	2.5 (1.6)
Acceptability (data from 22 mother–daughter dyads)		
Satisfaction^a^	Satisfaction with the PA classes	4.6 (0.8)
	Satisfaction with the program facilitators	4.7 (0.9)
	Satisfaction with program content and information delivered during sessions	4.8 (0.6)
	Rating of the overall experience in the program	3.7 (0.6)
Program recommendation^b^	Likelihood of recommending the program to a family member or friend	3.9 (0.3)

Abbreviation: PA, physical activity.

a Mothers responded on a 5-point Likert-scale, with higher numbers representing greater satisfaction.

b Mothers responded on a 4-point Likert-scale, with higher numbers presenting greater likelihood of recommending the program.

**Table 3:** Feasibility and Acceptability of Conmigo: Findings From Postintervention Qualitative Interviews With Mothers and Daughters (n = 32)

Feasibility and acceptability		
Program curriculum	Curriculum topics and structure meet the needs of families and align with their values. Favorite session topics included parenting and communication; dyads enjoyed completing weekly PA challenges together.	“[the challenges] were something nice and fun that I enjoy with my family; they were tasks that I could really enjoy and show at the end of the week.”—Mother “I like the parenting because I took parenting classes when she was little, but now she’s in a different stage and it’s so much more different than when she was little.”—Mother “SMART goals were helpful and I liked them, and communication was good.”—Daughter “Communication was a very important topic because when we started to talk about topics that I hadn’t talked about much with [daughter], it helped us have a better communication and connected us.”—Mother “Three of my favorites were nutrition because it showed how you can improve in like eating and stuff like that, and then I like the friendship one because it can also like help with people … and I’m stuck between communication and social media.”—Daughter
Overall satisfaction	Participants described enjoying program activities and being pleased by perceived improvements in their mother–daughter relationship and health behaviors; mothers consistently stated that they would recommend the program to others.	“It was a great program, great information, great physical activity, everything that we did. So I really enjoyed it.”—Mother “I really think this is a great program and that it helps a lot.”—Daughter “I feel like we’re gonna have these memories forever, it was a great time with my daughter.”—Mother “Overall, I liked everything.”—Mother “ I feel like it’s an overall pretty good program and there’s not much I would change about it.”—Daughter
Online delivery mode	While online delivery reduced barriers of childcare and transportation, most families noted that they would prefer in-person programming to connect with other families and better engage girls. Many families experienced occasional disruption in internet connection.	“I think people would really enjoy it more having it in-person … . It was interesting, but it would be more interesting, more interactive where you’re there.”—Mother “It was good, but I think it would be better if it was in-person.”—Daughter “But I did have a really hard time with my internet. A lot of times I had to shut it off and shut it down and bring it back up again … I think having the video, makes it worse. It gets clogged more.”—Mother “I think it would be better if it was in-person because you would get to interact more with other people and your mom.”—Daughter “It’s just convenient because if we have to go to the park for my son’s soccer practice, I was able to still participate being in two places at one time.”—Mother

Abbreviation: PA, physical activity.

**Table 4:** Conmigo Program Impact: Findings From Postintervention Qualitative Interviews With Mothers and Daughters (n = 32)

Program impact		
PA and diet	Dyads reported engaging in more PA weekly with an emphasis on going for walks together. Most dyads reported implementing small dietary changes most frequently, including additions of fruits and vegetables and substitutions of water for sugar-sweetened beverages.	“I’ve been keeping up with doing yoga or doing cardio on my own, and even if it’s like 20 min, it’s like some intense 20 min, so I’m still keeping up with that.”—Mother “I still have my goal of at least doing exercise two times a week. We walk mostly every day. We try to go every day, unless it rains … . It’s kind of a brisk walk cause we do go up terrain so it does get our heart rate up.”—Mother “And I started drinking water more and I started drinking less sugary drinks.”—Daughter “[Daughter] is a little bit more open to going out and doing some of the exercises and stuff, so I think it was good for her to learn that.”—Mother “We’ve been going for a walk and we’re going to keep doing that and I think we’re going to plan our schedule so we can fit everything in that we need to do.”—Daughter “It helped us a lot. My daughter was very encouraged to eat healthier, workout more and be more active. She constantly kept saying like today, she even asked me, ‘Mom can we go bike riding’ and I feel really happy about that, because she would usually just want to be on her tablet, watching TV, playing Barbies or play with the pets and not with me and now she kind of wants to do more stuff with me.”—Mother “We still eat vegetables and we’re not drinking sugary drinks, and so I think it really did help.”—Mother
Mother–daughter relationship and communication, parenting	Dyads reported spending more time together, improving mother–daughter communication, and having a healthier relationship.	“Building a little bit more of a relationship as a mother-daughter … sometimes I’m like ‘you know what not right now,’ you know ‘I’m so tired.’ And at least those two hours that we were there, we could discuss whatever the topic was.”—Mother “I have a connection now with my mom, that I didn’t have back then. So I think it’s worth it.”—Daughter “It helped me, like how to talk about difficult topics. It helped a lot because I didn’t know that.”—Mother “I think it has changed us … helped me to be more understanding and patient. She has also changed and she’s liked it too. So we are more connected now.”—Mother “Liked parenting topic since daughter is at a different stage in her life and things are different.”—Mother “Parenting styles was most helpful to learn because Mom learned the importance of giving reasons when she says ‘No.’”—Mother “Liked learning about others parenting styles and offering support because able to see how hers are different/similar to her parents growing up.”—Mother

Abbreviation: PA, physical activity.

## Key Points

- The Conmigo online mother–daughter physical activity intervention demonstrated feasibility with high fidelity and high satisfaction among Latina families.
- Despite positive engagement, daughters showed no significant improvement in self-reported or accelerometer-assessed physical activity.
- Improvements in maternal limit-setting behaviors suggest potential benefits for parenting practices that support physical activity.
